# In-Season Nutrition Strategies and Recovery Modalities to Enhance Recovery for Basketball Players: A Narrative Review

**DOI:** 10.1007/s40279-021-01606-7

**Published:** 2021-12-14

**Authors:** Jon K. Davis, Sara Y. Oikawa, Shona Halson, Jessica Stephens, Shane O’Riordan, Kevin Luhrs, Bridget Sopena, Lindsay B. Baker

**Affiliations:** 1 Gatorade Sports Science Institute, PepsiCo, Inc., 3800 Gaylord Parkway, Suite 210, Frisco, TX 75034 USA; 2 Gatorade Sports Science Institute, PepsiCo, Inc., Bradenton, FL 34210 USA; 3 Institute for Health and Sport (iHeS), Victoria University, Melbourne, Australia; 4 Department of Physiology, Australian Institute of Sport, Canberra, Australia; 5 ACT Academy of Sport, Canberra, Australia; 6 Gatorade Sports Science Institute, PepsiCo, Inc., Barrington, IL 60010 USA

## Abstract

Basketball players face multiple challenges to in-season recovery. The purpose of this article is to review the literature on recovery modalities and nutritional strategies for basketball players and practical applications that can be incorporated throughout the season at various levels of competition. Sleep, protein, carbohydrate, and fluids should be the foundational components emphasized throughout the season for home and away games to promote recovery. Travel, whether by air or bus, poses nutritional and sleep challenges, therefore teams should be strategic about packing snacks and fluid options while on the road. Practitioners should also plan for meals at hotels and during air travel for their players. Basketball players should aim for a minimum of 8 h of sleep per night and be encouraged to get extra sleep during congested schedules since back-to back games, high workloads, and travel may negatively influence night-time sleep. Regular sleep monitoring, education, and feedback may aid in optimizing sleep in basketball players. In addition, incorporating consistent training times may be beneficial to reduce bed and wake time variability. Hydrotherapy, compression garments, and massage may also provide an effective recovery modality to incorporate post-competition. Future research, however, is warranted to understand the influence these modalities have on enhancing recovery in basketball players. Overall, a strategic well-rounded approach, encompassing both nutrition and recovery modality strategies, should be carefully considered and implemented with teams to support basketball players’ recovery for training and competition throughout the season.

## Key Points

**Table:** 

Sleep, carbohydrates, protein, and fluids should be the foundational recovery components of the basketball player during the competitive season as these areas currently have the most scientific evidence supporting their efficacy.
It is recommended that basketball players consume 5–7 g/kg/day of carbohydrate to replenish muscle glycogen stores and 1.2–2.0 g/kg/day of protein, consumed throughout the day (i.e., every 4–5 h), at 0.31 g/kg per occasion, to support muscle recovery. Players should consider fluid composition and the rate of ingesting fluids after training and competition, especially when there is < 24 h between practices/games.
Basketball players should aim for a minimum of 8 h of sleep per night and should be encouraged to get extra sleep during congested schedules (back-to-back games) and after periods of extensive travel, especially when traveling over multiple time zones.
Future research is warranted with recovery modalities such as hydrotherapy, compression garments, cryotherapy, and massage with basketball players. Teams should look to practically incorporate these recovery modalities with an individualized approach during the season to promote players’ recovery.

## Introduction

Basketball is a high-intensity intermittent court-based team sport that involves rapid change in movement patterns, accelerations, and decelerations throughout a 32–48-min game, depending on the level of play [1, 2]. During a basketball game players will perform various movements including high-intensity sprinting, jumping, and shuffling with repeated transitions between offense and defense [3, 4]. Male and female basketball players have been reported to cover 5–6 km at average intensities above the lactate threshold and 85% of maximal heart rate [1]. Both aerobic and anaerobic metabolic pathways contribute to players’ energy requirements on the court [2]. Specifically, glycolysis plays a key role in meeting adenosine triphosphate (ATP) demands due to the high-intensity nature of the game [1, 2]. Given the high metabolic rates from the game and reliance on glycolysis, carbohydrates are the main fuel source for basketball players [5, 6]. Large sweat losses during training and competition have also been reported [7, 8], with individual sweat losses varying widely among players [7–9]. Protein is another important factor for post-game recovery in facilitating muscle repair and remodeling [10] due to the repeated eccentric muscle contractions during game play [1, 2]. Similarly, collagen supplementation may also be relevant because of the high volume of jumping involved in basketball [1]. Nutritional strategies focused on rehydration, muscle protein synthesis (MPS), and carbohydrate repletion are central to promoting recovery from training and competition [11].

The National Basketball Association (NBA) consists of 82 games during the season, with 41 of the games at home and 41 of the games on the road. The season can last up to 8 months if the team qualifies for playoffs, with a potential to play over 100 games in total. Given the repeated travel, with some games being on back-to-back days, the timing of games, with the majority ending late at night, the high game loads, and the multiple time zone changes, managing recovery from metabolic and psychological fatigue over the season is crucial. In recent years, the NBA has taken steps to reduce the number of back-to-back games to allow for more rest and recovery between games. Also, load monitoring has become an important topic around the league, with some teams strategically resting players during games. Few studies have investigated the relationship between game load and injury risk with basketball players [12–14]. One study suggests that game schedules may be a potential risk factor for game injuries in the NBA [13], while others have shown mixed results [12, 14]. Post-exercise recovery is not only important for the NBA player, but for players at all levels of competition, including other professional basketball leagues (e.g., NBA G-League, Women’s NBA, European leagues) and college and high-school competitions. Given the on-court demands and off-the court challenges with extensive travel, recovering from a game with nutrition strategies and recovery modalities becomes critical to prepare for the next competition, both mentally and physically, and to maintain high-level performance throughout the season.

Several papers have addressed the metabolic demands of the game [1, 2], monitoring fatigue [15], and the influence of air travel [16], but few reviews have focused on recovery strategies for basketball players that can be practically incorporated throughout the season to promote recovery. Therefore, the purpose of this review is to examine the literature on recovery modalities (e.g., sleep, hydrotherapy, cryotherapy, compression garments [CG], and massage) and nutritional strategies (e.g., carbohydrates, fluids, protein, collagen, and nutrition for travel) for basketball players and practical applications that can be incorporated throughout the season at various levels of competition. This review will not include micronutrients and supplements for promoting recovery since there is limited research with basketball players and previous papers have addressed this topic for team sport athletes in general [11]. Additionally, this review does not include an exhaustive view of all recovery modalities available to basketball players. Rather, the review will focus on five popular and commonly employed recovery modalities that have been examined in other team sports [17, 18]. Throughout the article “recovery” will be referred to in the context of in-season recovery strategies for basketball players. The goal is to help players recover from game to game regarding muscle function, repair, and soreness, along with perceptual fatigue so that performance outcomes and player readiness are optimized. This review will also discuss practical recommendations and applications that coaches and athletes can implement during the season.

## Sleep

Optimizing sleep is often regarded as the best recovery strategy available to athletes [19]. Sleep plays an important role in performance, cognitive function, energy metabolism, muscle repair, mood, and illness prevention [20]. In high-intensity intermittent team sports, such as basketball, where recovery may be limited due to congested schedules, protecting and improving sleep where needed is crucial. While published data on sleep characteristics in the highest levels of professional basketball are limited, research in sub-elite or development athletes as well as other team sport research can provide insight into many of the challenges associated with sleep in basketball players.

In a recent assessment of the sleep need of elite athletes, it was reported that elite male and female athletes subjectively report they require 8.3 ± 0.9 h of sleep to feel rested [21]. However, a majority of athletes (71%) fail to meet this need on most nights, with an average sleep duration of 6.7 ± 0.8 h. This results in a sleep deficit index of 96.0 ± 60.6 min. Basketball athletes had the latest wake times (07:54 ± 00:24 h:min) and the longest sleep durations (7.5 ± 0.4 h) when comparing across various sports (mean wake times 07:18 ± 00:48 h:min; mean sleep durations 6.7 ± 0.8 h).

Supporting the important role of sleep on performance in basketball, Mah et al. [22] reported improvements in sprint times, shooting accuracy, and reaction time in male collegiate basketball players after 5–7 weeks of sleep extension. Objectively measured sleep duration was increased by 110.9 ± 79.7 min after the athletes were provided with a goal of spending 10 h in bed each night.

Many elite team sport athletes complain of poor sleep after games, in particular night games, which may be compounded when subjected to congested schedules. Seventeen elite Australian female basketball players had their sleep assessed, using activity monitors, over two seasons, totaling 30 weeks [23]. Match schedule influenced total sleep time, with double-headers resulting in players experiencing an 11% reduction in total sleep time when compared to the regular match schedule (one game per round). Elevated adrenaline and noradrenaline concentrations have been found before and after night games, when compared to a rest day, in elite netball players [24]. Further, athletes who had a tendency towards a high trait arousal were more susceptible to sleep complaints following a night game [24].

Workload during training and competition may also influence sleep characteristics. Fox et al. [25] examined the impact of workload during training and competition on subsequent sleep duration and sleep quality in seven semi-professional male basketball players. Sleep onset time was significantly later following medium and high training and competition workloads compared with control nights. Time in bed and sleep duration were also significantly shorter following high training and competition workloads compared with control nights [25]. Similar findings were found in 38 elite Australian Rules Football players, where high maximal running speeds and player loads during training were negatively associated with objective sleep markers [26]. However, sleep/wake behaviors were not influenced by training load in 11 elite female adolescent basketball players [27]. In this study, players did not obtain the recommended 8–10 h of sleep per night on training days. However, sleep onset and offset times were later on rest days than training days, while time in bed and total sleep time were greater on rest days compared to training days. This variability in sleep and wake times was also found in elite rugby league players [28], and while data regarding the effects of increased variability on sleep quality are not available in athletes, data in the general population suggest that high intra-individual variability in bed and wake times may have a negative influence on sleep quality and wellbeing [29]. Therefore, the scheduling of training sessions should be as consistent as possible.

Training sessions should also not be scheduled too early in the morning so that training encroaches on players’ total sleep time available. Early morning training sessions have been shown to reduce total sleep time in elite swimmers [30] and in rugby players [31]. This can be particularly challenging with multiple games per week and the potential associated travel. However, avoiding unnecessarily early training sessions is likely of benefit to the players.

There is increasing interest in the role of sleep and its relationship to both injury and illness [32]. In a recent study examining over 500 netball athletes, injury and illness had significant bidirectional associations with sleep duration and quality [32]. Low sleep duration in the 48-h period prior was associated with increased injury risk (odds ratio [OR] = 0.91 ± 0.03), while "very poor" sleep quality (OR = 0.59 ± 0.02) or extremes of too little (< 5 h, OR = 1.01 ± 0.03) and too much (> 10 h, OR = 1.01 ± 0.03) sleep had bidirectional associations with an increased illness risk [32]. This early work suggests the potential for a relationship between sleep and illness and injury.

To optimize and improve sleep, basketball players may benefit from sleep monitoring, feedback, and education [20]. Avoiding excessive screen time [33] and inappropriate caffeine intake [34] and providing education regarding general good sleep behaviors may help protect sleep in a sport where optimal sleep may be somewhat challenging given certain schedules. Recent research has highlighted the protective effects of business class travel in elite athletes, due to an ability to obtain high-quality and long-duration sleep in a fully reclined position [35]. Monitoring and education has been shown to be useful in the short term; however, consistent and persistent messaging to athletes is likely important [36].

## Hydrotherapy

Hydrotherapy strategies such as cold-water immersion (CWI), contrast water therapy (CWT), and hot-water immersion (HWI) have become commonly used recovery methods for elite athletes. These strategies are utilized regularly by athletes from many different team sports to aid recovery after training or competition [37]. While there is now a reasonable amount of evidence to support the use of hydrotherapy for athletic recovery, there remains minimal research that is basketball specific. The majority of studies examining hydrotherapy recovery specifically with basketball athletes have focused on CWI [38–41].

CWI is typically performed as either a full-body or legs-only immersion in water ranging from 5 to 20 °C, for durations of 3–20 min, with immersions being performed either continuously or intermittently [42]. CWI aims to reduce body temperatures and blood flow, which lead to reductions in swelling, inflammation, and pain [43]. It has been suggested that 11–15 min of continuous immersion at 11–15 °C is optimal for reducing muscle soreness [44]. However, a recent study that compared the effect of continuous (12 min at 12 °C) and intermittent (4 × 2 min at 12 °C) CWI following training with basketball athletes found both protocols were equally effective at aiding the recovery of muscle soreness and jumping capacity [45]. Similarly, Delextrat et al. [39] found 5 × 2 min at 11 °C CWI benefited perceived recovery and countermovement jump performance following a single game of basketball. CWI has also been shown to have positive effects on recovery over multiple games, with 5 × 1 min at 11 °C enabling better maintenance of line drill performance compared to a control over a 3-day basketball tournament [41]. It has also been found that CWI (5 × 1 min at 11 °C) over a 3-day tournament results in decreased inflammatory and muscle damage biomarkers compared to the control group [40]. Only one study has investigated the chronic use of CWI over a season. It was found that regular use of CWI (5 × 2 min at 10.5 °C) led to improvements in isokinetic strength and rating of perceived exertion at the end of the season compared to a control [38]. Based on the body of evidence examining the benefits of intermittent CWI on perceptual, performance, and biomarker recovery following basketball training, matches, and tournaments, it may be recommended that teams use intermittent protocols rather than continuous protocols. Also, from a practical perspective, implementing intermittent protocols can also be more time effective when trying to accommodate a larger squad if limited facilities or space are available. Full-body water immersion is recommended where possible to enhance the impact of hydrostatic pressure and to expose a larger surface area to the cold stimulus to enable greater heat exchange to occur [34]. However, leg-only immersion can still be beneficial to recovery [36] and should be used when full-body immersion is not able to be achieved.

While CWI has been shown to improve performance following a single game, a multi-day tournament, and chronically during a season of basketball, there remains some contention around the chronic use of CWI due to recent findings that it can attenuate strength adaptations [46, 47]. CWI should be programmed in as part of a periodized plan; it is recommended that CWI should be prioritized during periods of intensified competition and avoided when strength adaptations are a priority. A recent study by Tavares et al. [48] showed that, when programmed appropriately, CWI can be regularly used during a preseason training phase without having a negative impact on strength adaptations. The effect of CWI on performance recovery and training adaptations is influenced by many factors, and it is recommended that practitioners use an individualized and periodized approach when programming sessions to optimize performance benefits and minimize risks of negatively impacting adaptations.

CWT is also a commonly utilized recovery strategy by team sport athletes [37, 49]. CWT typically involves alternating between CWI (5–20 °C) and HWI (≥ 36 °C) three to seven times, with a duration of 1–2 min per immersion [42]. CWT aims to enhance blood flow, remove metabolic waste, and reduce inflammation [49]. To our knowledge, there are no studies examining the effect of CWT in basketball athletes, despite it being anecdotally reported to be highly utilized by basketball athletes [50]. Protocol selection for CWT is largely based on anecdotal reports, and the optimal number and duration of rotations are still unknown. However, it has been shown that an equal ratio of hot to cold improves cycling time trial performance [51]. CWT has also been shown to reduce perceptions of pain after eccentric exercise [50] and to enhance the restoration of strength and power after muscle-damaging exercise [52]. CWT protocols are also time effective to implement with larger teams, with athletes apportioned between the cold and hot water.

Although HWI is another popular hydrotherapy method, it is less frequently compared to CWI and CWT [53]. HWI involves immersion in water ≥ 36 °C for 10–24 min [42]. The aim of HWI is to ease muscle tension and increase blood flow to assist in the removal of metabolic waste and increase nutrient delivery to and from the cells [54]. HWI has been shown to enhance the maintenance of neuromuscular performance after intense exercise [55] and enhance the recovery of isometric force compared to control after muscle-damaging exercise [56]. While these studies show positive impacts of HWI on athletic recovery, more research is required to fully understand the physiological and performance effects of HWI and how this might be applied in a basketball specific scenario.

In summary, based on the current research and knowledge surrounding the use of hydrotherapy for recovery, CWI has the greatest level of support both specific to basketball and generally across a variety of athletes. Regular use of CWI in season to help athletes manage the cumulative fatigue of frequent competitions, travel, and training is recommended. Utilizing CWI post-game can be logistically challenging when travelling, as not all facilities are set up with inbuilt pool facilities. However, there are many commercially available portable ice bath options. Portable ice baths can either be inflatable pools or large plastic tubs, which can be filled with water and ice or connected to chilling systems that cool the water to maintain desired temperatures. If travelling with portable ice bath equipment is not an option, the use of a bathtub at the team’s hotel can be a more practical solution. With appropriate planning, CWI can be implemented effectively regardless of the team’s location. Table 1 provides further recommendations and practical applications for using these modalities.

**Table 1:** Practical applications to implement and promote recovery modalities during the season for basketball players

Sleep	Hydrotherapy and cryotherapy	Compression garments and massage
Basketball players should aim for a minimum of 8 h of sleep per night Players should be encouraged to sleep longer or nap more frequently during congested schedules or after periods of travel Practitioners should be aware that workloads in both training and games may negatively influence night-time sleep Coaches should consider strategies that increase time available for sleep, in particular the timing of any training sessions or travel the day following a game Consistent training times may be beneficial to reduce bed and wake time variability Regular sleep monitoring, education, and feedback may aid in optimizing sleep in basketball players Avoid blue light exposure after night games or use a blue blocker on electric devices Consider establishing a designated, comfortable, and convenient rest/sleep area within the home arena or facility A practical and less invasive way to monitor sleep is through subjective evaluation (e.g., validated sleep surveys, questionnaires), although objective sleep monitoring may reduce bias	Cold-water immersion (CWI) •Regular use of CWI is recommended in season or at tournaments to help manage fatigue •Intermittent protocols are recommended as a time effective alternative to continuous protocols when trying to accommodate a large squad •Full-body immersion is preferable; however, leg-only immersion will still provide positive benefits •CWI is recommended when athletes are recovering from muscle damage or high body temperatures Contrast water therapy (CWT) •Implementing CWT can be time effective for larger teams with athletes apportioned between the cold and hot pools •CWT is recommended when athletes are recovering from general fatigue or tiredness Hot-water immersion (HWI) •HWI is recommended when athletes are aiming to ease muscle tension and relax •HWI should be avoided during the acute phase of soft tissue injuries Hygiene considerations for hydrotherapy •Athletes should avoid water immersion when they have open wounds •Athletes should not complete water immersion when they are unwell •Where large groups are moving through pools, water should be treated with a chlorine or disinfectant solution Travel considerations for hydrotherapy •If space is available, consider traveling with portable, inflatable hydrotherapy pools to implement CWI protocols •Inflatable tubs can be utilized by players at the hotel post-game or after a long evening of travel •Encourage players to create their own CWI tubs in hotel rooms during temporary periods of travel and extended hotel stays. This may be more achievable if 10–20 lb bags of ice are provided to the players Cryotherapy •Future research is warranted regarding cryotherapy and recovery measures with basketball players before this is implemented in a post-game routine	Compression garments •Lower-body compression garments (e.g., tights, socks) should be encouraged post-training or post-match •Wear garments for as long as it is comfortable post-exercise and including night-time sleep. However, do not let garments compromise quality of sleep •Caution is advised with standard-sized garments as anthropometric variations between players may lead to inadequate pressure •If possible, players should purchase custom-fitted compression garments that exert a minimum pressure of 14 mmHg •Compression socks should be worn during long-haul flights to minimize the risk of flight-related thrombosis Massage •Consider offering/implementing a sport massage for players the following day after night-time competition •The massage should be short in duration (i.e., 5–12 min) compared to a longer massage (i.e., 15 min or longer)

## Cryotherapy

Several studies have investigated post-exercise whole-body and partial cryotherapy in various sports [44–46]. Cryotherapy typically involves standing in a special chamber with temperatures ranging from − 110 to − 190 °C for 2–5 min [57–59]. This may involve one session or multiple sessions per day of cryotherapy over a period of consecutive days. A recent review by Rose et al. [58] assessing various laboratory and applied studies with athletes and physically active individuals reported that post-exercise muscle pain was reduced in 80% of studies following cryotherapy. Studies have also shown improvements with cryotherapy regarding muscle function (i.e., maximal voluntary contraction) and performance (i.e., counter movement jumps, running performance) following muscle-damaging exercise [60–63]. This means that participants were able to return to pre-exercise baseline measurements faster with cryotherapy compared to control groups. Additionally, studies assessing cryotherapy on promoting post-exercise recovery have shown reduction of systemic inflammation and lower concentrations of markers for muscle cell damage [63, 64]. However, not all studies have reported improvements in muscle damage, reduction in inflammation, or performance-related outcomes with cryotherapy after exercise [65–67].

Only one study has investigated cryotherapy and post-game recovery in basketball players [68]. Bouzigon et al. [68] assessed thermal sensation ratings (i.e., cold-perceived sensation) during 3 min of cold exposure at − 130 °C in 24 international-level male and female basketball players from the French national team competing at the European Championship. Partial cryotherapy (all body parts exposed excluding the head and neck) was performed every afternoon over a 2-week period. The results showed that a 3-min cryotherapy exposure was well tolerated by both male and female elite basketball players and can be used during a heavy competition or training period. The authors did report large inter-individual differences in thermal sensation ratings, primarily due to variation in body mass index among players. Specifically, attention should be given to female basketball players with a lower body mass index as they seem to be more sensitive to cold, which could potentially affect compliance with cryotherapy. Several studies have incorporated similar temperatures (− 110 °C) and exposure times (3 min) and have shown decreased muscle soreness [69] and enhanced eccentric muscle performance recovery [60].

In summary, future studies are warranted with basketball players to understand the influence cryotherapy has in promoting recovery. Although cryotherapy offers a quick solution to promote post-game recovery, the method is expensive and likely not available to basketball players at all levels of play. If players or teams have access and plan to implement cryotherapy at their facility, multiple exposures are recommended, at 3 min for each session, conducted immediately after and in the 2–3 days following competition, at temperatures ranging from − 110 to − 140 °C [58]. Implementing cryotherapy during travel is challenging for most teams. Cryotherapy is more likely to be implemented post-game while playing at home if teams have this available at their facilities.

## Compression Garments

CG have become a popular recovery tool in athletic populations [70–72]. These garments are designed to provide graduated external pressure to a limb, typically increasing in pressure from the distal to proximal portions of an arm or leg [73]. The application of pressure may serve to increase venous blood flow and lymphatic outflow, thus enhancing the removal of muscle metabolites, subsequently minimizing muscle damage and inflammation [70, 74]. Also, the external pressure may reduce the space available for swelling to occur, attenuating the inflammatory response and preventing further muscle damage [75]. Compression-induced reductions in muscle damage can serve to enhance subsequent performance [76–78] and limit perceptions of muscle soreness and fatigue [79, 80]. The effects of CG use for recovery from basketball are equivocal, with only a limited number of studies available [40, 41, 72]. However, given basketball places stress on the lower body through running and jumping activities, inferences could be made from research investigating the influence of CG on recovery measures (e.g., muscle damage, performance, psychological) involving similar lower-body activities [77, 80–82].

Only one study to date has investigated CG use post-exercise on measures of muscle damage in a basketball setting. Montgomery et al. [40] showed CG worn for 18 h post-basketball game had little benefit on any measures of muscle damage, including blood biomarkers (creatine kinase, fatty acid-binding protein, myoglobin, interleukin [IL]-6, and IL-10) and mid-thigh girth circumference. These results are in disagreement with research highlighting a reduction of muscle damage markers with CG use post-exercise [77, 82, 83]. Furthermore, a series of meta-analyses found CG use post-exercise is useful for reducing creatine kinase concentration [75, 84] and muscle swelling [75, 85]. A likely explanation for the lack of benefit reported by Montgomery et al. [40] and other compression research [79, 81, 86] is the exercise protocols may not have been intense enough to induce a sufficient degree of muscle damage [49, 79]. This is supported by a meta-analysis reporting CG use more beneficial following resistance exercise rather than endurance or running activities. Studies by Montgomery et al. [41] and Atkins et al. [72] showed that wearing lower-body CG for 18 and 15 h post-basketball exercise, respectively, did not enhance exercise performance recovery. Conversely, previous research has identified that post-exercise use of CG is beneficial in improving exercise performance recovery [77, 78, 81]. A potential reason for the lack of benefit for performance recovery observed in these basketball studies [41, 72] is insufficient pressure applied by the CG. For example, Atkins et al. [72] used pressure levels of 7–10 mmHg across the lower body, but > 14 mmHg is suggested to be effective for exercise recovery [76] and a pressure of > 20 mmHg is recommended to increase blood flow [87, 88]. Also, resting measures of lower-limb venous and muscle blood flow of male basketball players are reported to increase with various CG styles (i.e., tights, socks, and shorts) [89]. These findings suggest CG are an effective strategy to increase blood flow in basketball players, which is the underlying mechanism thought to be responsible for the benefits of compression for recovery [70, 74].

Ratings of perceived muscle soreness and fatigue are reported to be positively influenced by the use of CG post-basketball games [41] or the basketball simulation exercise test [72]. The use of CG throughout a 3-day basketball tournament reduced perceived muscle soreness and fatigue compared to a control group [41]. Similar results were observed in the study by Atkins et al. [72], where lower-body CG worn following exercise improved ratings of perceived muscle soreness. These findings are supported by reviews reporting moderate benefits of CG on reducing post-exercise muscle soreness [70, 84, 85]. The observed benefits on perceived muscle soreness may be due to increases in blood flow, decreases in inflammation, and reducing space for swelling to occur with CG use [72, 79]. However, since a prior belief in the benefit of CG can influence results [90], a placebo effect might be responsible for the CG-induced benefits on subjective measures of recovery [72].

Athletes frequently experience long periods of immobility (i.e., sitting) due to the travel commitments of away games or competitions. As a result, venous stasis and procoagulant activity are promoted [91]. Thus, an athlete's risk of developing venous thrombosis is exacerbated, particularly from long-haul air travel [92]. Further, the incidence of travel-induced complications may be higher in basketball players due to the association between leg length and venous thromboembolism risk [93]. A strategy frequently recommended to prevent travel-related thrombosis is CG [91]. The application of compression during long-haul air travel has been reported to have a positive effect on indicators of coagulation [94], the maintenance of countermovement jump performance [95, 96], muscle swelling [96], and subjective measures (e.g., muscle soreness, energy levels, alertness) [96, 97]. Although these findings are not specific to basketball athletes, CG may serve as a useful strategy to minimize the adverse effects of long-haul travel (i.e., jet lag, travel fatigue, and reduced mental and physical performance) [98].

In summary, the use of CG after exercise appears to be beneficial for enhancing the recovery process [74, 75, 77, 84], albeit there is limited research available in basketball players. Further research is needed to provide basketball-specific practical recommendations. In addition, due to the high use of upper body actions in basketball (e.g., shooting, passing, overhead catching), more research is needed to determine how compression of the upper limbs may influence a basketball player’s recovery. However, as CG are inexpensive and non-invasive and likely to benefit perceived recovery after exercise [41, 72] and minimize effects of jet lag and travel fatigue [95, 96], they can easily be implemented as a recovery strategy for basketball players. Players should implement CG post-game prior to leaving the locker and specifically before traveling by bus or plane. Table 1 provides further detail for practical applications and recommendations for using CG to promote recovery.

## Massage

Massage is a recovery modality frequently undertaken by team sport athletes [99]. Several studies have evaluated massage to aid recovery in basketball players. Delextrat et al. [39] had 16 basketball players (eight men and eight women) complete a 30-min massage immediately after a competitive match. Players were selected from four top ranking teams in the University Premier League. Perceptual measures were assessed immediately and 24 h post-competition. Physical performance measures, countermovement jump and repeated-sprint ability, were assessed in a non-fatigued state to set baseline measures prior to the game and then 24 h after the competitive match. The results showed a significantly lower perception of fatigue in the legs immediately after the massage, but no difference in perceptual or performance measures 24 h post-match.

In a follow-up study, the authors showed that incorporating stretching with a massage immediately after a competitive basketball game resulted in significantly lower perception of leg soreness and significantly improved repeated sprint performance compared to massage alone [100]. This means that players were able to return to non-fatigue baseline faster when massage and stretching were implemented immediately after competition. Mancinelli et al. [101] showed massage incorporated after exercise improved vertical jump displacement and perceived soreness in college basketball and volleyball players [101]. However, not all studies have shown massage to promote post-exercise recovery of jump performance [39, 102] or sprint performance [39, 103].

A recent study of university basketball players showed that massage increased heart rate variability and parasympathetic activity and reduced sympathetic activity [104]. Massage was applied during half-time of a simulated basketball game in which players received 10 min of a traditional Thai massage or rested. Although these markers could potentially indicate the basketball player is less fatigued [15], it is unclear whether these outcomes are harmful or beneficial at halftime of a game, and how they relate to on court performance or recovery post-game. While novel, the ability to include a massage during half-time of a game would be challenging, and it is likely players would need to focus on fueling, hydration, and the coach’s game time adjustments for the second half of play. Future investigations should follow-up on these results and understand the applications of including this type of massage technique on post-game recovery markers.

Several studies assessing massage from a post-exercise recovery perspective have shown it to reduce delayed onset muscle soreness [39, 101, 105]. These results should not be understated as the notion of helping players feel better may be beneficial in promoting psychological recovery throughout the season. In a recent meta-analysis on the influence of massage on recovery, Poppendieck et al. [99] concluded that (1) a short massage time is more effective (i.e., 5–12 min) than a longer massage (i.e., 15 min or longer), (2) massage should be included immediately post-exercise, and (3) massage appears more effective after intense exercise. The shorter massage duration has practical applications for players receiving treatment the following day in the team’s facility and makes it practical for the team to implement massage with a number of players prior to morning practice and game film session. However, implementing massage immediately post-game especially for professional basketball players may be challenging. Players may have to fulfill media obligations post-game or may be required to get on the team bus or flight immediately after the game. Nonetheless, massage should be implemented into the recovery routine of basketball players to help promote recovery even if the main benefit is from a psychological perspective [99, 106]. Table 1 provides practical applications and recommendations for using massage with basketball players.

## Nutrition: Fluids, Carbohydrates, and Protein

### Fluids

Body water plays an integral role in thermoregulation through the dissipation of heat by sweat evaporation during exercise [107]. Sweating rates vary considerably among players, ranging from ~ 0.5 to 2.5 L/h [108, 109] depending upon body size, exercise intensity, and amount of playing time. If fluid intake is insufficient to replace sweat losses, a body water deficit, or hypohydration, will accrue [110]. During recovery, players should replace any remaining fluid deficit accrued through exercise and consume adequate fluids throughout the day to begin their next training session or game euhydrated. In general, observational studies suggest that basketball players are at a relatively low risk of significant hypohydration during games, likely due to the ample access to fluid and breaks in play [110]. However, players with heavy sweating rates and who receive minimal substitution breaks may be at a higher risk of accruing significant hypohydration. In addition, congested schedules and travel, particularly to warmer areas, may make it difficult to drink appropriately (e.g., volume, rate, type of fluid ingested) throughout the day. Accordingly, studies suggest that it is common for basketball athletes, especially male players, to begin practice or competition with urine concentrations that may be indicative of hypohydration (urine specific gravity ≥ 1.020) [7, 111]. With these considerations in mind, fluid intake strategies after exercise should be based on individual needs. In addition, adequate all-around fluid intake before and during practices/games lessens the need for aggressive post-exercise rehydration, which allows athletes to focus their attention on other nutrition needs or recovery modalities.

Athletes who are hypohydrated at the onset of exercise may be at risk for detriments to performance [112]. A few studies have induced hypohydration via exercise and/or heat exposure prior to measurement of basketball-specific skills. Baker et al. [113] found that, in 17- to 28-year-old male players, progressive dehydration from 1 to 4% was associated with a progressive decline in basketball skill performance, such as various on-court sprinting and shooting drills. Similarly, Dougherty et al. [114] found that 2% hypohydration impaired 12- to 15-year-old boys’ performance during basketball-specific sprinting, lateral sliding, and shooting drills. In these studies, hypohydration established prior to simulated play was associated with increased ratings of fatigue and impaired attention [115], but had no effect on ratings of perceived exertion [113, 114]. Louis et al. [116] reported that 2% hypohydration decreased 3-point shooting performance in seven of nine elite basketball players and increased ratings of perceived exertion compared to when they were euhydrated. Similarly, perceived exertion was higher when adolescent athletes began a 30-min series of basketball drills 2.5% hypohydrated compared to when they were permitted water intake (1.0% hypohydration) prior to performance testing, but there were no significant differences in measures of basketball performance [117]. Taken together, this literature suggests that beginning a game in a hypohydrated state may negatively impact the athlete either perceptually (fatigue) or objectively (skill) and as such, the utilization of proper post-exercise hydration strategies is important for recovery and for subsequent performance in basketball players.

If the body water deficit from practice or competition is small or the duration between bouts is prolonged (≥ 24 h), it is not necessary to implement an aggressive rehydration strategy, as consuming foods and fluids as usual is likely sufficient to re-establish euhydration. However, if the duration between exercise bouts is short, an athlete should consider a more regimented rehydration plan. Rapid and complete rehydration is especially important if participating in a practice session or game within 24 h [118]. In this case, strategies for adequate hydration after exercise should consider the volume, rate, and composition of fluid intake between games/training [119, 120].

The first step in determining appropriate volumes of fluid intake after exercise is hydration monitoring via measurement of body mass changes. Objective hydration assessment is important because athletes tend to poorly conceptualize their sweat losses during exercise [121]. For example, Thigpen et al. [122] found that the error in self-estimating sweat losses was high (~ 71%) among male and female National Collegiate Athletic Association Division II players. These findings highlight the importance of including objective measures such as body mass change to assess hydration status and determine the volume of fluid intake needed to restore euhydration. Further, the use of multiple hydration assessment methods (e.g., body mass, urine specific gravity, and thirst) is likely a more accurate indication than one method alone in determining whether players are drinking enough throughout the day [123].

To account for continued sweating after cessation of exercise as well as obligatory urine loss to excrete metabolic wastes, athletes should consume more than 100% of the accrued fluid deficit during recovery. For example, Shirreffs et al. [124] found that 6 h after fluid ingestion, net fluid balance was completely restored only when fluid intake was greater than fluid lost during exercise. However, there was no clear advantage to drinking 200% compared to 150% fluid replacement. The rate of fluid ingestion after exercise also impacts fluid retention. Since rapid ingestion of fluids after exercise may result in a temporary increase in diuresis [98], the rate of ingestion should be tailored to optimize fluid retention. Jones et al. [102] induced 2% hypohydration in healthy young men and then had participants consume water equal to 100% of fluid loss in either a bolus (100% consumed in 1 h) or in a metered pattern (12.5% of fluid loss every 30 min for 4 h). Urine output was 40% lower in the metered ingestion pattern and resulted in 27% greater fluid retention than a single bolus of water consumption. Similar results comparing metered versus bolus drinking were found with a carbohydrate/electrolyte/protein beverage [125]. Thus, athletes should ingest fluids in smaller amounts over a longer period of time to avoid rapid reductions in plasma osmolality and aid in fluid retention.

Finally, the composition of fluids consumed during recovery also significantly impacts rehydration following basketball play. Consumption of plain water during rehydration can decrease plasma sodium concentration and plasma osmolality, resulting in an increase in urine production and a decrease in thirst [126]. The addition of sodium increases serum sodium concentration and stimulates renal water reabsorption, which helps to restore plasma volume and whole-body fluid balance [119]. In turn, ingesting fluids with sodium promotes better fluid retention after exercise-induced hypohydration than electrolyte-free fluids [124, 127, 128]. Another mechanism to promote fluid retention is by increasing energy density (e.g., inclusion of protein and/or ≥ 10–12% carbohydrate), which slows fluid delivery to the circulation, thereby delaying urine losses after fluid ingestion. In particular, the increased energy density as well as the clotting of casein in milk-based products delays gastric emptying [129, 130]. Practitioners should consider the duration between exercise bouts when deciding the most appropriate composition of a fluid replacement beverage for basketball players. Milk or other energy-dense carbohydrate/protein-based drinks are effective rehydration beverages when there are several hours between exercise bouts. However, when rapid rehydration is needed and delayed gastric emptying could cause practical issues (gastrointestinal distress), energy dense fluids such as milk may not be suitable. In these situations, athletes should drink up to ∼ 1.5 L of a sodium-containing fluid (with relatively low energy density) for each kilogram of body fluid deficit to promote fluid retention without delaying fluid delivery to the circulation. Further, regarding the rehydration efficacy of other common beverage components consumed throughout the day, moderate caffeine (up to 400 mg) does not impact fluid balance [131], but drinks with > 2% alcohol can impair rehydration [120].

### Carbohydrates

The prolonged stop and go nature of basketball requires dependence on both the anaerobic and aerobic energy systems and as a result, an increased reliance on carbohydrates as a primary fuel source [132]. Importantly, aerobic metabolism acts as the primary contributor to ATP regeneration between sprints and increases reliance on intramuscular glycogen stores throughout a basketball event [132]. Thus, basketball players should aim to adequately restore skeletal muscle glycogen during recovery from prolonged competition and training by adhering to guidelines for carbohydrate intake for team sport athletes [132].

Beginning exercise in a state of glycogen depletion can negatively affect subsequent performance in basketball players [133]. As was eloquently shown by Michalczyk et al. [133], introduction of a 4-week low-carbohydrate diet in basketball players resulted in a significant reduction in total work capacity as measured by a Wingate anaerobic test. After 1 week of carbohydrate re-loading, total work capacity was not significantly different from baseline values, suggesting that low muscle glycogen had reduced anaerobic capacity [133]. Although this was the only study to assess the effects of a low- or high-carbohydrate diet in basketball players, the findings are congruent with others showing that high-carbohydrate diets compared to lower-carbohydrate diets prolong exercise performance [134, 135] and increase time spent in high-intensity exercise [136]. Thus, it is imperative that basketball players ingest sufficient carbohydrate after training and games to limit decrements in performance associated with low muscle glycogen status at the beginning of subsequent exercise bouts.

Current recommendations suggest that team sport athletes consume 5–12 g/kg of body mass of carbohydrate per day for recovery from moderate to very high-intensity exercise. The lower end of the range would be appropriate for moderate exercise or athletes who do not get much playing time, and higher ranges would be appropriate for very high-intensity exercise or athletes who play a lot of game minutes [137]. However, studies suggest that carbohydrate intake is generally lower than that recommended for team sports athletes [138, 139]. For example, a study by Nikić et al. [140] administered a food frequency questionnaire to elite junior basketball players (15–16 years) and found that daily carbohydrate intake was below 6 g/kg/day in 56% of the participants. Similarly, Baker et al. [141] examined 24-h carbohydrate intake in elite adolescent athletes and found that 84% of male athletes and only 42% of female athletes were consuming the recommended 5–7 g/kg/day of carbohydrate. Interestingly, Schröder and colleagues [142] observed that despite a generally high total energy intake overall compared to other team sport athletes, elite Spanish basketball players failed to meet recommended carbohydrate guidelines and consumed on average only 4.5 g/kg/day. Reviews of dietary habits of basketball athletes suggest that carbohydrate intake does not decline over the course of a season; rather, carbohydrate consumption is consistently lower than recommended from the start of the season [139]. Little is known about the carbohydrate intake habits of basketball athletes immediately post-exercise. Baker et al. [141] found that only 68% of male athletes and 43% of female athletes met the adequate intake (1.0–1.2 g/kg/h) for post-exercise carbohydrate intake for the hour following exercise. When broken down by sport, only 38% of basketball players (seven males and one female) consumed adequate carbohydrate during the 1 h following exercise [141]. These results should be interpreted with caution, however, as accurate and reliable dietary intake measurements are often difficult to obtain.

Although data are limited, the literature collectively suggests that elite basketball players do not consume adequate carbohydrate to achieve daily intake recommendations for team sport athletes and thus may be at risk for beginning games or training in a state of insufficient glycogen repletion. Future studies should examine long-term dietary intake patterns of basketball players, and in particular, whether players are consuming adequate carbohydrate in the acute post-exercise period to replenish muscle glycogen stores.

### Protein

The physical requirements of a basketball game are intermittent in nature and composed of elements involving sprinting, maximal jumping, and rapid changes in direction [2]. These movements present a unique physiological stress that requires a combination of eccentric (braking), isometric (planting), and concentric loading (propulsive acceleration) [143], ultimately resulting in increased muscle damage [144]. Exercise and protein ingestion are the two main anabolic stimuli responsible for increasing rates of MPS, where ingestion of protein in close temporal proximity to exercise results in greater stimulation of MPS than either stimulus independently [145]. To date, no study has directly investigated the effects of basketball play on rates of MPS. However, given that resistance exercise [146, 147], endurance exercise [148, 149], and concurrent exercise [150] have been shown to augment rates of MPS, it could be reasoned that, following game play, an increase in MPS would also be observed in basketball players.

To facilitate the repair and remodeling of skeletal muscle, bone, and connective tissues, athletes require a greater amount of dietary protein than what is currently recommended to the general population [151]. Current guidelines by the National Academy of Medicine recommend that protein be consumed at 0.8 g/kg/day for all adults over the age of 18 [152]. However, a joint position statement by The Academy of Nutrition and Dietetics, Dietitians of Canada, and the American College of Sports Medicine [153] recommends that athletes aim to consume protein at 1.2–2.0 g/kg/day, to support recovery. Specifically, protein is recommended on a per occasion basis for athletes at 0.31 g/kg [154], to be consumed every 4–5 h, in an effort to evenly spread protein intake throughout the day and achieve maximal rates of MPS more frequently [155]. Evidence from elite basketball players suggests that most athletes are consuming protein within the range as recommended per day [140–142]; however, there are no data to date that have specifically characterized protein intake patterns in basketball athletes. Gillen et al. [156] monitored the dietary intake patterns for protein-containing foods in over 550 well-trained endurance and team sport athletes and found that, on average, both male and female athletes consumed protein at the recommended levels for athletes (1.5 and 1.4 g/kg/day, respectively). However, the authors noted that only 42% of athletes consumed > 20 g of protein at breakfast and only 64% consumed > 20 g of protein at their mid-day meal [156]. These data suggest that the majority of athletes are likely achieving maximal rates of MPS with only their dinner meals and that altering protein distribution to a more even pattern throughout the day may be a beneficial strategy to augment recovery and adaptation. Given that these data were cross-sectional, future research should aim to determine performance outcomes from skewed or balanced protein intakes in elite athletes.

Another consideration to optimize protein intake to benefit skeletal muscle health is the consumption of protein prior to sleep. Research into the factors influencing muscle protein anabolism in the overnight period has been limited thus far to the use of casein protein, a fraction of milk protein. Unlike whey protein that is rapidly digested and absorbed, casein protein coagulates upon ingestion when mixed with gastric acid, resulting in a more protracted release of amino acids [157, 158]. It is these distinguishing factors that make casein an attractive option to consume prior to evening periods of sleep wherein individuals will likely not be consuming protein or participating in significant physical activity (the two main anabolic stimulators to MPS) for a prolonged period of time. Indeed, Res et al. [159] measured muscle protein turnover during sleep in healthy young males and showed that consuming 40 g of casein protein prior to sleep (with a bout of resistance exercise performed earlier in the day) significantly increased MPS and improved net protein balance compared to the pre-sleep ingestion of an isocaloric placebo control. Similarly, Trommelen et al. [160] observed a greater whole-body net protein balance in healthy, young individuals consuming 30 g of casein protein prior to sleep compared to a placebo control. To date, no studies have examined the chronic effects of pre-sleep protein ingestion on skeletal muscle health or performance. However, protein ingestion prior to sleep may serve as an effective strategy for athletes to consume protein more evenly throughout the day.

Finally, in conjunction with total daily protein intake and protein distribution, protein quality is crucial to optimizing muscle protein anabolism. Dietary protein quality is assessed by the Food and Agricultural Organization of the United Nations using the Protein Digestibility Corrected Amino Acid Score or more recently, the Digestible Indispensable Amino Acid Score. In both calculations, proteins are evaluated by digestion and by the amount of the limiting essential amino acid within the protein source [161]. Typically, animal sources of protein (meat, eggs, dairy) are considered to be high-quality protein sources and also contain high levels of the essential amino acid leucine [162], whereas plant protein sources are often classed as lower quality protein due to low levels of lysine, leucine, and methionine [163]. Vegetarian diets, and the reliance on plant-based sources for protein, are increasing in popularity [164]. Indeed, approximately one-third of athletes at the Delhi 2010 Commonwealth games reported practising a vegetarian diet or avoiding meat consumption [165]. Importantly, there is little evidence that athletes consuming a vegetarian diet require more protein than athletes consuming an omnivorous diet [166]. However, given the differential amino acid profiles between most plant-based protein sources and animal-based protein sources, it is important that vegetarian and vegan athletes are attentive to consuming foods from a variety of sources to ensure that adequate amounts of all essential amino acids are consumed. Notably, leucine is the only amino acid that has been shown to independently stimulate MPS [167]. Plant sources of protein typically contain a lower leucine content than animal-based protein sources [163] and as such typically result in lower rates of MPS when measured acutely following exercise [168]. Further, when compared in absolute quantities during prolonged resistance training, animal-based protein (cow’s milk) has been shown to be superior at inducing increases in skeletal muscle hypertrophy compared to chronic consumption of plant-based proteins (soy beverage) despite the beverages being isonitrogenous [169]. Importantly, when plant-based protein sources are consumed in sufficient quantities such that total leucine is within the threshold range to maximally stimulate MPS (1.7–2.4 g) [170], adaptations to exercise with plant protein supplementation have been shown to be similar to supplementation with animal-based protein sources [171]. Thus, it is hypothesized that adequate plant protein ingestion to match animal protein intake post-exercise would result in similar benefits to skeletal muscle health and performance. However, to date, no studies have investigated the effects of chronic consumption of plant-based diets in elite athletes.

### Collagen

It is well established that athletes require elevated protein intakes to optimize MPS for skeletal muscle health [172]; however, much less is known regarding nutritional requirements to optimize connective tissue health. Connective tissue health is of particular relevance to basketball players due to the high volume of jumping and intermittent sprinting required in game play. Collagen provides the structural foundation for connective tissue and is the most abundant protein in the body [173]. Connective tissue refers not only to tendons and ligaments, but also skeletal muscle collagen, which helps to transmit force throughout the length of the muscle [173]. Augmenting muscle stiffness has been shown to benefit sprinting speed where greater stiffness is associated with maximal running velocity [174]. Similarly, and particularly relevant to basketball, there is also a positive and direct relationship between muscle tendon stiffness (which contributes to skeletal muscle stiffness) and jump performance [175]. In turn, basketball training often contains a plyometric component in an effort to increase connective tissue stiffness and augment sprinting speed and jump performance [176].

Augmenting collagen deposition or collagen synthesis results in increases in muscle, tendon, and ligament stiffness [173]. Very few studies have examined the effects of nutrition on in vivo connective tissue protein synthesis, and no studies have evaluated connective tissue protein synthesis following basketball play. Previous literature directly examining connective tissue synthesis is limited to resistance exercise models [177–179] and thus may not be entirely applicable to basketball athletes. However, these studies collectively suggest that unlike skeletal muscle, rates of muscle collagen synthesis are not elevated with the ingestion of essential amino acids or whey protein in the absence of exercise. However, it does appear mechanical loading can induce increases in collagen protein synthesis. Resistance exercise has been shown to augment rates of collagen synthesis in both perimysal collagen (skeletal muscle collagen) and tendon collagen [180, 181].

Recent evidence indicates that although unresponsive to essential amino acids and whey protein, connective tissue may be responsive to the provision of supplements derived from collagen such as collagen peptides and gelatin [182]. Collagen peptides and gelatin are incomplete protein sources (as they lack the essential amino acid tryptophan. rendering them ineffective at stimulating MPS [179]); however, both contain high proportions of the amino acids glycine and proline, the primary amino acids composing the structure of connective tissues [183]. Indeed, Shaw and colleagues [182] provided participants with 15 g of vitamin C enriched gelatin, 1 h prior to 6 min of continuous rope-skipping. The authors found that levels of amino-terminal propeptide of collagen I (P1NP), a marker of collagen synthesis, were double that of the placebo control group [182]. Similarly, although not statistically significant, Lis and Baar [184] observed an increase in serum P1NP in athletes supplemented with either gelatin or collagen peptides, 1 h following jump-rope exercise. These findings highlight the potential for collagen-derived protein supplementation to benefit connective tissue stiffness (via increased collagen synthesis) when consumed in close temporal proximity to jumping exercise and perhaps in turn greater explosive power in basketball players over time. Of note, glycine and proline are non-essential amino acids, meaning that athletes are capable of producing them in sufficient quantities and are not required to be consumed through diet to contribute to the synthesis of new proteins [162]. Thus, it is unclear as to the mechanisms driving an increase in collagen synthesis with collagen peptide or gelatin supplementation or whether consuming a complete protein source, such as whey, would result in similar increases in P1NP as shown in the aforementioned studies.

### Nutrition for Travel

**Fig. 1:**
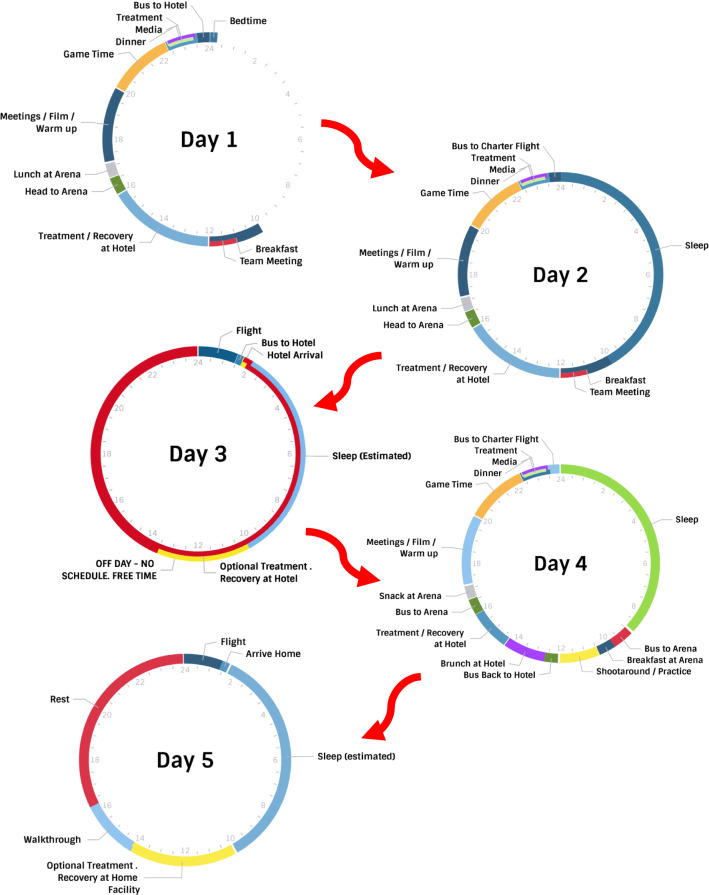
A practical example of a 5-day cycle that professional players face while travelling on the road

Collegiate and professional basketball players encounter the challenge of frequent travel, which presents some obstacles with respect to proper nutrition for optimal recovery. For instance, half of the regular season games in the NBA are away games, sometimes resulting in travel for 1–2 weeks. Figure 1 provides an example travel schedule of what professional players face on the road. For collegiate players, around 40% of their games may be on the road, and they could be away for 4–5 days in the middle of the school year. Air travel traversing multiple time zones has been shown to negatively impact power, agility, speed, and reaction time [95, 185, 186]. These decrements in performance have been reported in athletes accustomed to habitual overseas air travel on long-haul flights [186]. Disturbances in performance are evident upon arrival and can persist up to 24 h post-flight, or longer depending on the number of time zones crossed [95, 185, 186]. Performance decrements have been linked to disruptions in circadian rhythms, which contribute to feelings of jet lag, with symptoms including decreased alertness, insomnia, gastrointestinal distress, fatigue, headaches, impaired sleep, and loss of concentration and motivation [187–189]. For players in the NBA, the main disturbances in performance occur when traveling from the East Coast to West Coast [190, 191]. Roy and Forest reported that from 2010 to 2015, NBA teams traveling westward had a significantly lower winning percentage (36.2%) compared to teams traveling eastward (45.4%) [190]. Steenland and Deddens [191] also reported that NBA teams traveling from west to east performed better, scoring 4 more points per game compared to teams traveling from East Coast to West Coast. However, these studies did not account for the quality of the teams in the study [190, 191]. It has been suggested that performance is diminished from westward travel due to playing at a time players would likely be resting or preparing to sleep [190, 191]. It is therefore important to consider practical strategies that can reduce jet lag and help the player perform while traveling. Strategies reducing jet lag to adapt to the new time zone include light exposure to advance or delay shifting the circadian rhythm, exogenous melatonin intake, benzodiazepines, altered meal timing and composition, and adjusting to a new time zone prior to arrival [189, 192]. For the purpose of this section, nutritional strategies such as meal timing, meal composition, timing of caffeine consumption, catering, and practical strategies to incorporate fluid and macronutrient recommendations while traveling will be addressed. A more comprehensive discussion on nutrition and travel may be found elsewhere [189, 193–195]. Although it is beyond the scope of this review to discuss micronutrients and supplements, the authors would like to acknowledge the role of cherries and other phytonutrients in the management of sleep and soreness in team sports [196, 197]. From our experience, we have seen these micronutrients and supplements used by basketball teams, especially at the professional level, usually in juice extract form, but they are also incorporated into food menus and drink smoothies. The reader is referred to the following reviews for further inquiry [196–198].

Dietary strategies through nutrient timing and meal composition have been proposed to reduce symptoms of jet lag by enhancing adaptation of circadian clocks [199–202]. Animal models and human clinical trials support the notion that the composition and timing of meals can induce entrainment of the peripheral circadian clock (i.e., influence faster circadian rhythm adjustment to a new time zone) [199–201, 203, 204]. However, these studies have employed methods that are unrealistic for athletes, such as a time restricted feeding pattern (i.e., 24-h food deprivation protocol prior to feeding) [199–201, 203] or alternating high (no caloric limit) and low caloric intake days (limited to 800 kcal) [204]. Players should be encouraged to focus on foundational nutritional strategies, mentioned previously, with fluids, carbohydrates, and protein and practical applications for implementation (Tables 1 and 2) to promote recovery during travel whether via air or bus [193, 195].

**Table 2:** Practical applications to implement nutritional strategies for basketball players during the season

Carbohydrates	Fluids	Protein	Travel considerations
Daily intake requirements: 5–7 g/kg of carbohydrate per day	If sweat loss for the given practice or game is known, players should aim to consume 1.0–1.5 L of fluid for each 1 kg of body mass deficit if short rest between sessions; otherwise drink ad lib with meals/snacks if ≥ 24 h between session	Consume 1.2–2.0 g/kg/day in order to support recovery, including optimizing rates of muscle protein synthesis	There is limited evidence to support specific nutritional recommendations to reduce symptoms of jet lag with air travel
Consume carbohydrate with meals and have snacks with protein regularly throughout the day	When there is a relatively short break between training bouts, consume beverages and/or snacks with sodium during the recovery period to help replace sodium lost in sweat and promote fluid retention (limit diuresis)	Protein is recommended on a per occasion basis for athletes at 0.31 g/kg, to be consumed every 4–5 h in an effort to evenly spread protein intake throughout the day	Follow personalized nutrition recommendations for macronutrients and fluids to promote recovery after competition is recommended during air travel
Acute recovery	If possible, fluids should be ingested in a metered fashion (i.e., small amounts over 3–4 h rather than one large bolus) to avoid rapid reductions in plasma osmolality and aid in fluid retention	Consume 30–40 g of protein prior to sleep to promote recovery	Create a room-service food guide based on the hotel room-service menu to help the players make informed decisions
If players have > 8 h between competitions, then consume 5–7 g/kg (daily requirement) of carbohydrate per day	Players can assess their own hydration status by monitoring sensations of thirst and urine color throughout the day	15 g of collagen peptides or gelatin with vitamin C may support collagen synthesis; however, future research is warranted regarding the use of collagen peptides or gelatin to support recovery	If working with the hotel’s food service staff, a menu specifying high-carbohydrate foods, high-protein foods, produce, and fluids should be provided
If players have < 8 h before the next competition, consume 1.0–1.2 g/kg of carbohydrate every hour for 4 h to help replenish muscle and liver glycogen stores			Pack snack and fluid options while traveling by air or bus
			At hotels, a snack station within a banquet room or a room on a player floor can encourage continuous feeding throughout the day thereby optimizing glycogen replenishment and muscle protein synthesis for recovery
			Practitioners should investigate restaurant options around the hotel in advance that offer a healthy menu selection and outline well-rounded meal choices to guide players
			Practitioners should consider caterers that frequently provide meals to the arena when providing a post-game meal to players. The experience of these caterers can make the process easier and more efficient for the post-game meal

Another important aspect to consider while traveling is the timing of caffeine consumption. Caffeine has been shown to delay circadian melatonin rhythm when consumed 3 h before bedtime [205]. Considering the majority of NBA games are played at 6 p.m. or later, players should not consume caffeine post-game as this may contribute to the interruption of post-game sleep. It is also important to educate players who do consume caffeine to manage their intake to aid in the management of jet lag and fatigue while traveling. Caffeine has been shown to help alleviate some symptoms related to jet lag by reducing daytime sleepiness and could potentially improve resynchronization of the circadian system [188, 195, 206]. If players consume caffeine prior to the game, they should use the minimally effective dose (~ 3 mg/kg) rather than the large doses (6 mg/kg) associated with early studies of caffeine and performance, particularly for the evening games [207, 208].

From a practical perspective, strategically planning menus in advance to enhance recovery presents a challenge during travel. It is therefore important for athletes to plan ahead and pack non-perishable items to help meet individual macronutrient and fluid needs to enhance recovery. For instance, in transit to and from games, it is important to provide fluids, snacks, or possibly meals depending upon the amount of time it takes to reach the destination. Convenience foods such as granola bars, popcorn, baked chips, jerkies, yogurt, and fresh fruit, either separately or in combination, are some healthy options that are higher in carbohydrate, and players should also pack a complete source of protein. For professional basketball players who may fly charter or private plane, in-flight meals might be provided and can include a wider variety of options such as cooked vegetables and lean animal proteins. Regardless of the mode of transportation, planning is imperative to promote recovery post-game while traveling.

In addition, the team’s hotel plays a critical role in providing food during trips. Teams typically arrive one to two nights prior to the game, and the players may rely heavily on the hotel food service staff to meet their fueling needs. The team’s dietitian can create a food guide based on the hotel room service menu to help the players make informed decisions. Furthermore, if budgets are available, the hotel can provide team meals in the banquet rooms of the hotel. If working with the hotel’s food service staff, a menu specifying high carbohydrate foods, high protein foods, produce, and fluids should be provided. The team’s dietitian can create a static menu or menu guideline that can be sent to every hotel, or they can create a detailed menu based on the hotel’s banquet menu. In addition, strategic food placement within the banquet rooms and consistently in all of the hotels throughout the year can help guide the player on what foods to include on their plates as well as create a more efficient flow. For instance, always placing high carbohydrate foods such as potatoes, pastas, and rice in the front of the room where the players enter can help emphasize the importance and likelihood that players choose these foods. In addition, breakfast during morning meetings and a pregame meal at the hotel typically encourages the players to begin fueling earlier in the day and leading up to departure for the arena. Further, keeping snack options on-hand during travel or a snack station within a banquet room or on the players’ floor can encourage continuous feeding throughout the day, thereby optimizing glycogen replenishment and MPS for recovery.

Aside from hotel food, sometimes restaurants may be the best option. However, there might be cases, especially at the collegiate level, where teams are not staying at a hotel with a restaurant or the hotel restaurant may be closed after returning from a late-night game. Therefore, although not optimal, it may be necessary to develop nutritious options from fast-food menus to give to basketball players. Professional players may have the ability to venture out on their own and dine-in at a restaurant, or the entire team may decide to eat out. A restaurant might also be able to cater a meal in one of the hotel banquet rooms. Regardless, the team practitioner can influence the choices a player makes when choosing foods from a restaurant menu. Planning ahead of time on how to obtain nutritious meals post-game is crucial, whether it be from a non-hotel restaurant or the ability to have a catered-in meal.

To maintain consistency throughout the season, foods provided for home games should also be provided during away games. Therefore, teams may utilize travel bins to pack non-perishable food items. In addition, the arena’s primary food service provider may be able to provide and deliver perishable foods such as fruit, breads, and certain spreads. For post-game recovery nutrition, there may be certain caterers that frequently provide post-game meals to arenas. The experience of these caterers can make the process easier and more efficient as well as create more peace-of-mind with food safety. There may also be destinations that are known for certain cuisines which can make the experience more enjoyable for the players during travel. Either way, contacting the arena’s representative can help provide insights into catering options for teams. If local caterers are difficult to find, local grocery store chains are a good alternative to provide catered meals and snacks. In addition, making the post-game meal convenient and mobile is important since players may need to consume the meal on the bus or plane or at the hotel. Ultimately, because of the demanding schedules, it is imperative that proper nutrition on the road is emphasized just as much as it is during home stretches. Remaining as consistent as possible on the road as at home will help enhance recovery and readiness throughout the season.

## Conclusions

Basketball players face multiple challenges to in-season recovery, as training loads and busy travel schedules limit opportunities for rest between games. Therefore, players must be intentional in their recovery strategies (Tables 1 and 2). Protein, carbohydrate, and fluid are foundational nutrition components and should be emphasized throughout the season for home and away stretches to promote recovery. Teams should plan in advance for catering meals at hotels and during air travel for their players. Special consideration must also be given to supporting sleep education and sleep hygiene while considering practice schedules to accommodate sleep and rest throughout the season. Although more research is needed in basketball players, recovery modalities should not be discouraged since promising results have been shown, especially for CWI. Recovery modalities are typically studied in isolation to understand how they promote recovery after training or competition, but in reality, players and team personnel apply these modalities in combination (i.e., CG, hydrotherapy, massage). Further, personalized implementation of hydrotherapy should be considered, as this can offer the players a choice (CWI, CWT, HWI) while still utilizing best practices.

It is important to take a bespoke approach regarding personalization and prioritization of recovery strategies based on the individual’s playing time, their hydration and nutrition needs, and muscle soreness and fatigue level, among other factors. It should also be acknowledged that some of the recovery modalities discussed may have limited applicability to high-school or small colleges (Division II and III) because of resource availability. However, the high-school or small college athlete should still be able to incorporate several key recommendations to enhance recovery through sleep and nutrition. Overall, a strategic well-rounded approach, encompassing both nutrition and recovery modalities should be carefully considered and implemented to support the basketball player’s recovery throughout the season.
